# Did giraffe cardiovascular evolution solve the problem of heart failure with preserved ejection fraction?

**DOI:** 10.1093/emph/eoab016

**Published:** 2021-06-11

**Authors:** Barbara Natterson-Horowitz, Basil M Baccouche, Jennifer Mary Head, Tejas Shivkumar, Mads Frost Bertelsen, Christian Aalkjær, Morten H Smerup, Olujimi A Ajijola, Joseph Hadaya, Tobias Wang

**Affiliations:** 1 Department of Medicine, Harvard Medical School, Boston, MA, USA; 2 Department of Human Evolutionary Biology, Harvard University, Cambridge, MA, USA; 3 Division of Cardiology, David Geffen School of Medicine at UCLA, Los Angeles, CA, USA; 4 Department of Public Health and Primary Care, University of Cambridge, Cambridge, UK; 5 Zoobiquity Research Initiative at UCLA, Los Angeles, CA 90024, USA; 6 Brentwood School, Los Angeles, CA, USA; 7 Copenhagen Zoo, Frederiksberg, Denmark; 8 Department Biomedicine, Aarhus University, Aarhus, Denmark; 9 Department of Cardiothoracic Surgery, Copenhagen University Hospital, Rigshospitalet, Copenhagen, Denmark; 10 UCLA Cardiac Arrhythmia Center, David Geffen School of Medicine at UCLA, Los Angeles, CA, USA; 11 Molecular, Cellular and Integrative Physiology Program, UCLA, Los Angeles, CA, USA; 12 Zoophysiology, Department of Biology, Aarhus University, Aarhus, Denmark

**Keywords:** cardiovascular, left ventricular hypertrophy, heart failure with preserved ejection fraction, resistance, comparative

## Abstract

The evolved adaptations of other species can be a source of insight for novel biomedical innovation. Limitations of traditional animal models for the study of some pathologies are fueling efforts to find new approaches to biomedical investigation. One emerging approach recognizes the evolved adaptations in other species as possible solutions to human pathology. The giraffe heart, for example, appears resistant to pathology related to heart failure with preserved ejection fraction (HFpEF)—a leading form of hypertension-associated cardiovascular disease in humans. Here, we postulate that the physiological pressure-induced left ventricular thickening in giraffes does not result in the pathological cardiovascular changes observed in humans with hypertension. The mechanisms underlying this cardiovascular adaptation to high blood pressure in the giraffe may be a bioinspired roadmap for preventive and therapeutic strategies for human HFpEF.

## INTRODUCTION

### Bioinspired medicine

Evolutionary medicine has emerged as a powerful lens through which we can better understand the nature and origins of human pathology. Evolutionary perspectives can also accelerate biomedical innovation. The emerging field of biomimicry can serve as a source of novel approaches to human pathophysiology [1]. Bioinspired medicine (or biomimicry) recognizes the physiologic differences across species as a source of solutions to challenges encountered by the evolutionary ancestors of extant individuals.

Biodiversity arises as organisms facing different challenges and opportunities evolve into phenotypes which are better aligned with their environments. Thus, the physiologic adaptations of other species may be conceived of as solutions to these challenges and optimized responses to opportunity. As such, contained within the biodiversity of physiologies of other species on the planet may be countless solutions to physiologic challenges to human health.

Outside the field of medicine, biomimicry has been a source of solutions for problems from biofouling [2] to architectural instability [3]. Natural phenomena can serve as an inspiration for the creation of structures, products, services, and solutions. Among the earliest and most well-known commercial applications of biomimicry was the invention of Velcro by Swiss engineer George de Mestral, which was inspired by Mestral’s observations that the burrs of the burdock plant stuck to his clothes and dog’s fur [4]. Since the inception of Velcro in the early 1940s, there has been a steep increase in biomimicry research for a wide range of applications. While invertebrates have proven to be a rich source of bioinspired insights, from surgical glue inspired by the natural adhesive of bivalve mussels [5] to mosquito-inspired microneedles and microprobe implants [6, 7], vertebrates have also inspired a broad range of innovations in locomotion, flight technology, defense systems, and many other fields [8–10]. In addition to technological innovations, biomimicry has accelerated the development of products directly related to human health, such as antibacterial and sunscreen activities from the gel-like red sweat of the hippopotamus [11], antimicrobial surfaces that mimic shark skin [12], robotic limb design for prosthetics based on marine species [13], and bioinspired methods of drug delivery [14].

Comparative medicine involving traditional animal models with vulnerability to human pathology has provided many insights. However, limitations associated with traditional animal models are fueling efforts to find novel approaches [15]. Bioinspired approaches which draw parallels between pathological conditions in humans and analogous, non-pathological systems in other animals have the potential to yield insights and innovations traditional methods have not.

### Evolved adaptations: finding solutions for a leading cause of heart failure

Cardiovascular disease (CVD) is the leading cause of death in the USA, killing one person every 37 s [16]. Heart failure (HF)—the leading reason for hospitalization in patients over 65 years of age in the USA—is a chronic progressive form of CVD that is characterized by the heart’s inability to pump sufficient blood to meet the requirements of the body. HF syndromes are often grouped together based on whether the left ventricular (LV) ejection fraction (EF) is preserved (HFpEF) or reduced (HFpEF) [17]. Heart failure with preserved ejection fraction (HFpEF) accounts for half of all human HF diagnoses and has an estimated 5-year survival of only 38% [18]. While advances in both pharmacologic and device-based therapies over the past four decades have significantly lowered mortality and morbidity in patients with reduced LV ejection fraction [19], despite significant research investment, similar progress has yet to be made in treating HFpEF.

### HFpEF pathophysiology

The mammalian heart displays a high degree of plasticity in order to adapt to changes in environmental conditions and increased workload. Cardiac remodeling—the ability of the heart to adapt to increased workload demand by undergoing changes in size, shape, structure and function—is a vital adaptive feature of the mammalian heart [20]. In humans, ventricular hypertrophy, a form of cardiac remodeling, may develop as a physiologic (non-pathologic) response to exercise, development, and/or pregnancy [20, 21]. Pathologic ventricular hypertrophy develops in response to volume or pressure overload with an eccentric form (↓ in LV wall thickness, ↑ in LV cavity size) typically linked to volume overload and concentric hypertrophy (↑ in LV wall thickness, ↓ or no change in LV cavity size) associated with pressure overload [20, 22, 23].

Pathologic concentric LV hypertrophy in humans commonly occurs as a physiologic response to longstanding poorly controlled systemic hypertension and/or significant aortic stenosis. As predicted by the Law of Laplace (i.e. the tension within the wall of a sphere is directly proportional to the thickness of the sphere), the individual’s LV thickens to alleviate increased wall stress as afterload (the resistance the heart must overcome to eject blood during systole) increases with rising blood pressure or progressively decreasing aortic valve outflow area [24].

As the LV thickens to contain wall stress, the relative size of the ventricular cavity decreases [20–23, 25, 26]. Pathologic cardiac remodeling is associated with numerous pathophysiologic changes including increased cell death and diffuse interstitial collagen fiber deposition [20, 25, 26]. Fibrosis and other related processes underlie the increased ventricular stiffness (i.e. reduced compliance) which contributes to the impaired exercise tolerance and other clinical symptoms of HFpEF [20, 27, 28].

### A proposed model of resistance to HFpEF

Despite the cadre of bioinspired examples from outside the field of medicine, there have been few efforts to apply biomimicry to key challenges in human health including CVDs such as HFpEF. While multiple conditions (e.g. obesity, coronary artery disease, diabetes, and chronic kidney disease) are associated with HFpEF, systemic hypertension usually plays a central role in the pathology [29]. A lack of suitable animal models has been identified as one source of limited therapeutic innovation in HFpEF [30]. The identification of nonhuman animals with evolved resistance to the adverse effects of hypertension on the human myocardium could spark much needed innovation in research. We hypothesize that evolved cardiovascular adaptation in giraffe protect its ventricles from the pathologic changes associated with systemic hypertension leading to HFpEF in humans.

### Giraffe cardiovascular system

**Figure 1.**
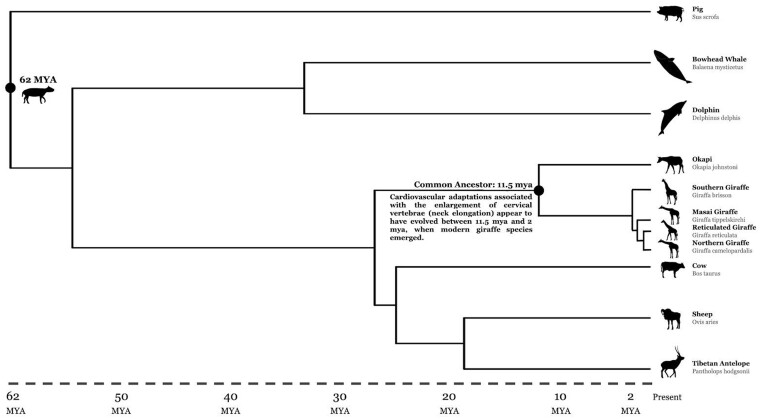
Evolutionary divergence of the giraffe from the okapi and other mammals. Created using the Interactive Tree of Life [31].

The giraffe, *Giraffa* spp., is the world’s tallest animal and stands at over 5.5 m tall. Giraffes diverged from their closest extant relative, the okapi, approximately 10–12 million years ago (Fig. 1) [31]. While the okapi is similar in body shape, it lacks the ironically long neck of the giraffe. The giraffe’s long neck is hypothesized to have evolved to allow greater access to high foliage, to enhance predator detection, and to influence sexual selection [32].

In giraffe, the lengthening of the neck over the course of development and general somatic growth substantially increases the vertical distance between the heart and the brain. This vertical distance may exceed 2.5 m/s. The need to maintain adequate cerebral perfusion with progressive neck lengthening during development leads to increasing systolic blood pressures; systolic blood pressures in healthy adult giraffe fall between 200 and 300 mmHg at the level of the heart [33]. Healthy adult giraffe blood pressures are more than twice that of humans and other mammals as a function of body mass [34]. While substantially hypertensive by non-giraffe mammalian standards, these pressures are not only normal for giraffe, they are crucial for the hemodynamic performance of the species [33–38].

**Figure 2.**
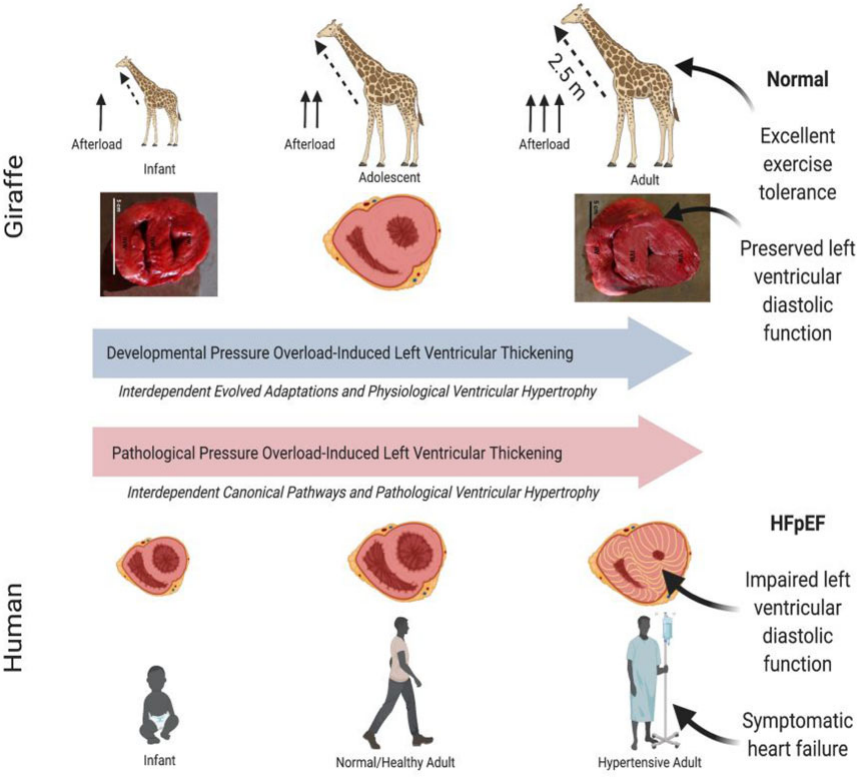
Comparison of the response of the left ventricle of the giraffe to increasing hypertension over the course of development vs. the ventricular response to chronic hypertension in humans. In both cases, hypertension leads to thickening (i.e. hypertrophy) of the left ventricular wall. Hypertension-induced left ventricular thickening (LVT) in humans leads to cardiac pathologies such as fibrosis, and commonly heart failure with preserved ejection fraction (HFpEF). However, developmental pressure-induced LVT in growing giraffes does not compromise exercise capacity, which is an important adaptation this prey species. HFpEF, heart failure with preserved ejection fraction.

The thickness of the giraffe ventricle at birth is comparable to what has been observed in other newborn mammals [35]. However, as the neck lengthens and blood pressure increases to maintain cerebral perfusion, the LV and interventricular walls develop concentric thickening (i.e. hypertrophy) [33, 35]. As predicted by the Law of Laplace, the giraffe’s LV thickens to alleviate increased wall stress as afterload increases concomitantly with neck length [24]. The gross LV morphology of the giraffe shares many characteristics with that of other mammals. However, the relationship between LV wall thickness and cavity size in modern giraffes does not follow the typical patterned relationship observed in other mammals [35, 39, 40]. Most notably, the volume of the LV cavity in the adult giraffe is smaller than expected relative to other species, especially in comparison to the relative thickness of its ventricular wall [33, 34]. A comparable ratio in humans is typically associated with hemodynamic impairment and clinical symptomology, especially during exercise (Fig. 2).

### Are cardiovascular adaptations in giraffes a naturally occurring model of resistance to heart failure with preserved ejection fraction (HFpEF)?

While concentric LV hypertrophy in humans is associated with cellular and subcellular changes leading to increased ventricular stiffness, reduced exercise tolerance, and HFpEF, giraffe cardiovascular physiology does not follow this pattern. Giraffe appear to have evolved an adaptation protecting hypertrophic ventricle from these changes and from progression to HFpEF [25].

Predation risk may be the basis of the selective pressure underlying this adaptation. Giraffe are a prey species and to evade capture and death they must be able to flee predators at speeds of up to 60 km h^−1^ [41]. Thus, their survival, and ultimately fitness, depend on maintaining maximal exercise capacity in order to escape predators. If the increased afterload associated with increased neck length and concentric LV hypertrophy also induced the myocardial changes, which compromised exercise capacity (as is seen in humans with HFpEF), the fitness benefits of the increased neck length might be counterbalanced by increased risk of predation. In humans with HFpEF, increasing heart rates reduce relative diastolic filling times which leads to increased pulmonary pressures and HF symptoms. During flight from predators, increased myocardial oxygen demand contributes to rising heart rates and reduced diastolic filling times without apparent adverse effects on pulmonary pressures. Although hemodynamic measurements of giraffes exercising at maximum capacity are not presently available, a 1966 study that measured the cardiovascular responses of wild East African giraffes running to avoid capture recorded heart rates of up to 170 bpm via radiotelemetry [37].

**Figure 3.**
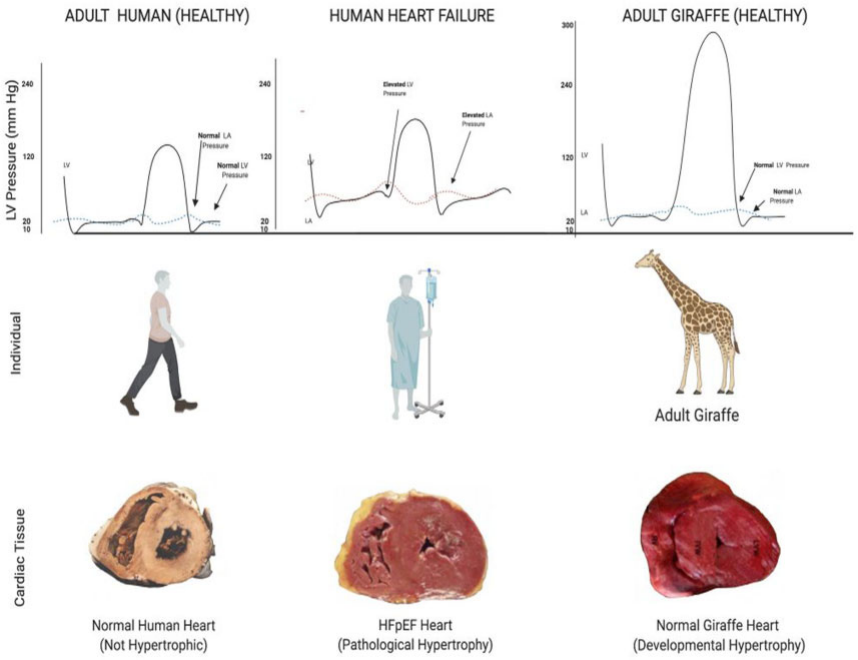
Comparison of gross ventricular anatomy in the healthy human adult heart, human heart failure, and healthy adult giraffe hearts and their relationships to left atrial and ventricular pressure. Despite significant pressure-induced left ventricular thickening (LVT), giraffe cardiac pressures do not display the elevated left atrial and ventricular pressures observed in humans with severe hypertension-induced LVT. Giraffe ventricular pressures adapted from Smerup *et al*. [33]. HFpEF, heart failure with preserved ejection fraction.

Giraffe cardiovascular physiology has particular salience for human HFpEF. The giraffe heart appears to be an example of a mammalian heart in which pressure-induced concentric ventricular thickening does *not* appear to reduce exercise capacity as is the case with pressure-induced concentric ventricular thickening and HFpEF in humans [28, 42]. We hypothesize that evolved adaptations in the giraffe myocardia prevent elevation in LV diastolic pressures that are observed in humans with HFpEF and magnified with exercise-associated tachycardia. Figure 3 compares the advanced hemodynamic consequences of pathological hypertension-induced hypertrophy seen with HFpEF in human with the adaptive physiological concentric LV hypertrophy that we hypothesize exists in the normal adult giraffe heart.

### Potential mechanisms

The cellular and subcellular processes that protect giraffe hearts from the adverse consequences of systemic hypertension and pressure-induced LV thickening are largely unknown. However, several candidate mechanisms are providing new insights in to these evolved adaptations. For example, cardiac fibrosis—a pathological process associated with systemic hypertension and HFpEF in humans, appears to be relatively suppressed in giraffe despite comparable levels of ventricular thickening [34]. Likewise, our preliminary data from reviewing 136 necropsy reports suggests a reduced propensity for myocardial fibrosis in the giraffe relative to humans and other mammalian species [43].

Consistent with our observations, reduced fibrosis in giraffe myocardia may be linked to differences in the amino acid sequence of the ACE protein [32, 44], as well as recently identified mutations in fibroblast growth factor receptor-like 1 (FGFRL1) [32, 45]. Notably, the FGFRL1 protein sequence in giraffe appears to be highly divergent in comparison to a diverse array of other mammals, with seven amino acid substitutions in a region that is crucial for FGF binding. In addition, a comparison of the giraffe genome to that of its closest living evolutionary relative, the okapi, identified 70 genes with ‘multiple signs of adaptation’ that were not observed in other eutherian mammals [32], five of which are found within the developmental pathways that lead to cardiac fibrosis [43].

In the recently published study by Liu *et al*. [45], mouse *FGFRL1* was edited to contain the seven amino acid substitutions of giraffe *FGFRL1* using CRISPR-Cas9 technology. The mutant mice with giraffe-type *FGFRL1* exhibited improved heart function and significantly less fibrosis in cardiac and renal tissues than wild-type mice in response to infusion with angiotensin II, indicating a role for FGFRL1 in suppressing fibrosis in the physiological setting of hypertension. Furthermore, the potential roles of micro RNAs in the post-transcriptional regulation of *ACE*, *ACE2*, *FGFRL1* and other relevant genes during cardiac remodeling further underscore the need to elucidate the underlying mechanisms of different cardiac phenotypes [23, 46]. Lastly, other genes involved in the regulation of fibrosis, as well as processes contributing to diastolic impairment in humans (i.e. autonomic regulation, neuroendocrine function, and myocardial innervation), could also play a role in the cardiovascular adaptations and unique exercise capacity of the modern giraffe. Precise characterization of the mechanisms underlying the giraffe heart’s resistance to the adverse effects of chronic pressure overload may yield important insight for preventing and treating HFpEF in humans.

## RECOGNIZING EVOLUTIONARY ADAPTATIONS AS A SOURCE OF THERAPEUTIC INNOVATIONS

The giraffe’s unique physiology has long been a source of fascination to biologists and physiologists. Goetz and Keen—two of the first scientists to gather concrete physiological data on the giraffe—noted that giraffes exhibited ‘high’ blood pressures by human standards [47]. The resistance of the giraffe cardiovascular system to orthostatic changes via shifts in neck position and the ability of its renal system to withstand high arterial pressures have also received extensive attention over the last 65 years [36, 37, 40, 48–56]. While earlier studies established thick ventricular walls in the giraffe [36], more recent studies on the unique cardiac adaptations of the giraffe heart to chronically high afterload have focused on the physiological and cellular underpinnings, such as myocardial architecture, cellular structure, and hemodynamics [19]. Importantly, Smerup *et al*. [33] demonstrated that ejection fractions, diastolic ventricular pressures and measures of LV wall stress remained in the ‘normal’ range in comparison to other mammals. Furthermore, normal diastolic pressures would not be predicted in a morphologically comparable human ventricle.

The existence of a mammalian cardiovascular system in which ventricular thickening from pressure-overload does not reduce diastolic relaxation or elevate cardiopulmonary pressures suggests that models of resistance to human cardiovascular pathologies may have evolved spontaneously in other species. Non-pathological cardiac remodeling during somatic growth in the giraffe also focuses attention on developmental pathways and related regulatory systems as potential approaches to HFpEF in humans.

Given the importance of pressure-induced physiological LV thickening in the giraffe and other species-specific cardiovascular characteristics for human health, why are these connections relatively unexplored? One factor has been the limited extent to which physicians perceive the natural world as a source of inspiration for complex human pathophysiology. Veterinarians and wildlife biologists are trained in the core discipline of comparative physiology, which seeks to emphasize both differences between species and the importance of elucidating the underlying mechanisms of how animals interact with and adapt to their environment. Yet, modern medical education does not traditionally include broad instruction on the diverse range of high-performance physiologies of other species. Greater collaborative interactions between physicians, veterinarians, animal physiologists and wildlife biologists would increase the likelihood that biomedical investigators could identify ‘solutions’ to challenging human pathophysiologies in the natural world.

Rudolf Virchow, the father of modern pathology, observed that, ‘Between human and animal medicine there is no dividing line’ [57]. Despite Virchow’s early insight, the separations between human, comparative, and veterinary cardiology persist. The lack of communication between these research fields impedes innovations to the detriment of human CVD. As physicians and investigators increasingly perceive biodiversity in the natural world as a source of insight for clinical medicine, bioinspired solutions to the most challenging cardiovascular issues may emerge.
